# On the Ability of Perfluorohexane Sulfonate (PFHxS) Bioaccumulation by Two *Pseudomonas* sp. Strains Isolated from PFAS-Contaminated Environmental Matrices

**DOI:** 10.3390/microorganisms8010092

**Published:** 2020-01-09

**Authors:** Alessandro Presentato, Silvia Lampis, Andrea Vantini, Flavio Manea, Francesca Daprà, Stefano Zuccoli, Giovanni Vallini

**Affiliations:** 1 Department of Biological, Chemical and Pharmaceutical Sciences and Technologies (STEBICEF), University of Palermo, 90128 Palermo, Italy; alessandro.presentato@unipa.it; 2 Department of Biotechnology, University of Verona, 37134 Verona, Italy; stefano.zuccoli@studenti.univr.it (S.Z.); giovanni.vallini@univr.it (G.V.); 3 Regional Agency for Environmental Prevention and Protection of Veneto (ARPAV), Regional Laboratories, 37135 Verona, Italy; andrea.vantini@arpa.veneto.it (A.V.); flavio.manea@arpa.veneto.it (F.M.); francesca.dapra@arpa.veneto.it (F.D.)

**Keywords:** bioaccumulation, bioremediation, emergent pollutants, PFASs, PFHxS, *Pseudomonas* sp., short-chain PFASs, xenobiotics

## Abstract

PFASs (perfluoroalkyl and polyfluoroalkyl substances) are highly fluorinated, aliphatic, synthetic compounds with high thermal and chemical stability as well as unique amphiphilic properties which make them ingredients in a range of industrial processes. PFASs have attracted consideration due to their persistence, toxicity and bioaccumulation tendency in the environment. Recently, attention has begun to be addressed to shorter-chain PFASs, such as perfluorohexane sulfonate [PFHxS], apparently less toxic to and more easily eliminated from lab animals. However, short-chain PFASs represent end-products from the transformation of fluorotelomers whose biotic breakdown reactions have not been identified to date. This means that such emergent pollutants will tend to accumulate and persist in ecosystems. Since we are just learning about the interaction between short-chain PFASs and microorganisms, this study reports on the response to PFHxS of two *Pseudomonas* sp. strains isolated from environmental matrices contaminated by PFASs. The PFHxS bioaccumulation potential of these strains was unveiled by exploiting different physiological conditions as either axenic or mixed cultures under alkanothrofic settings. Moreover, electron microscopy revealed nonorthodox features of the bacterial cells, as a consequence of the stress caused by both organic solvents and PFHxS in the culturing substrate.

## 1. Introduction

Per- and poly-fluorinated alkyl substances (collectively known as PFASs) represent a large family of chemicals, which includes more than 3000 fluoro compounds of anthropogenic origin, use, and disposal, as these organofluorides are not naturally found in the environment. PFASs are characterized by a single and/or multiple aliphatic chain, where all the carbon (C) atoms (per-) or some of them (poly-) are bound to fluorine (F) instead of hydrogen (H) atoms, forming the fluoroalkyl moiety (C_n_F_2n+1_^−^) to which carboxylate, phosphonate, sulfonamide, sulfonate, and alcohol head groups are linked [1]. Due to the chemical and thermal stability of the perfluoroalkyl moiety, as well as its water, oil, and stain repelling properties [2,3,4], these substances have been applied in several industrial fields, including the production of aqueous film-forming foam (AFFF), metal plating, textiles, leather and upholstery, aviation hydraulic fluid, semiconductor manufacturing, to name a few [5,6,7,8]. Due to the misuse of PFASs and their release in almost all environmental niche worldwide—even in the remote Arctic region—these compounds have reached, over time, alarming concentrations. For instance, perfluorooctane sulfonic acid (PFOS) was first demonstrated to occur in wildlife [9], while Hansen et al. (2001) revealed how PFOS and perfluorooctanoic acid (PFOA) were present in human blood coming from biological supply companies [10]. Thus, persistence and bioaccumulation tendency of certain PFASs began to be of emerging concern so that PFOS and PFOA were added to the list of persistent organic pollutants (POPs) at the Stockholm Convention in 2009 [11,12]. Moreover, under the pressure of the US Environmental Protection Agency (US EPA), 3M stopped producing PFOS in 2002, while DuPont phased out PFOA in 2005, being these two perfluorochemicals no longer produced in the USA.

We are now witnessing a shift towards shorter perfluoroalkyl chemicals which manufacturers are relying on for commercial purposes, as ultrashort—(C = 2–3) and short—(C = 4–7) chain PFASs represent an alternative to long ones (C > 7) [8,13,14]. However, the physical-chemical features of C_4_–C_6_ PFASs are not as good as those of long-chain PFASs, determining the need for a much larger amount of short-chain PFASs to achieve similar performances with the respect of long-chain fluoroalkyl compounds [5]. Moreover, short-chain PFASs (e.g., perfluorobutane (PFBS) and perfluorohexane (PFHxS)) possess a much higher water solubility and lower proficiency to adsorb to the organic matter than long-chain ones [14,15]. Although these alternative PFASs seem not to exhibit worrisome bioaccumulation potential and toxicity [16,17], they represent nevertheless end transformation products of fluorotelomers that tend to persist in the environment, being many of them currently either unknown or unidentified, since these chemicals are covered by trade secrecy or are manufacturing waste [7]. The high environmental mobility of short-chain PFASs, as well as the lack of comprehensive knowledge about their fate into the different ecosystems [18], have generated concern regarding their long-term and undefined effects and meanwhile, the need for sound and efficacious approaches for the reclamation of areas contaminated by these emergent pollutants.

Among the most used short-chain PFASs, PFHxS is a fully fluorinated alkyl compound that has been largely used as an efficient surfactant in place of both PFOS and PFOA due to its physical-chemical stability, however, increasing to a critical extent the environmental concentration of this short-chain PFAS [19,20]. Thus, in 2017, PFHxS, together with its salts and related compounds, has been identified as potentially hazardous chemical and added—under the EU REACH Regulation—to the list of very high concern substances (SVHC), because of its recalcitrance to both abiotic and biotic degradation as well as its enhanced bioaccumulative behavior, and, therefore, the longer half-life of PFHxS with the respect of both PFOA and PFOS [21].

The present study was driven by the need to fill knowledge gaps on the possible interactions between microorganisms and short-chain PFAS, gaining information on how autochthonous bacteria isolated from contaminated environmental matrices (i.e., soil and groundwater) can face the presence of PFHxS as a stressor. All this by even considering the rising alert about the diffuse contamination of short and long-chain perfluoroalkyl compounds in the main Italian river basins (i.e., Arno, Brenta, and Po), which are impacted by industrial discharges from polymer manufacturers, tanneries and textile industries, with the latter identified as a prominent source of short-chain PFASs [22]. Thus, axenic cultures of two different bacterial isolates—belonging to the *Pseudomonas* genus—exposed to PFHxS under alkanotrophic (i.e., octane, octanol, and a mixture of ethanol/octane as the only source of carbon and energy) conditions to elicit biotic cometabolic reactions were investigated to unveil the influence of different bacterial cell physiology (namely: growing and resting (i.e., metabolically active yet non-growing) cells) on the actual removal of PFHxS from the growth media over a short period of incubation. A similar experimental approach was adopted to test the PFHxS bioaccumulation potential of both isolates as a mixed culture. Finally, electron microscopy technique was applied to shed light on the potential effect of organic solvents alongside PFHxS exposure onto bacterial cell morphology and structural features.

## 2. Materials and Methods

### 2.1. Sample Collection and Microbial Enrichment Cultures

Soil and groundwater matrices deriving from an industrial site—located in the Veneto region (Italy)—highly contaminated by PFASs were provided by the regional agency for prevention and protection of the environment of Veneto (ARPAV).

To enrich the autochthonous biota present in the environmental matrices, 25 g of soil were dissolved in 250 mL Erlenmeyer flask containing 100 mL of defined medium (DM) [23], which was amended with yeast extract (YE; 0.01% *w*/*v*; Sigma-Aldrich^®^, Merck Life Science S.r.l., Milan, Italy) and 200 ng L^−1^ of a PFAS mixture dissolved in methanol containing sodium perfluorohexanesulfonate (PFHxS), sodium perfluorooctanesulfonate (PFOS), perfluorobutanoic acid (PFBA), perfluorohexanoic acid (PFHxA), perfluorooctanoic acid (PFOA), perfluorononanoic acid (PFNA), perfluorodecanoic acid (PFDA), perfluoroundecanoic acid (PFUdA), and perfluorododecanoic acid (PFDoA) (Wellington Laboratories Inc., Chemical Research 2000 S.r.l., Rome, Italy). In the case of contaminated groundwater, 10 mL of ten times (10×) concentrated DM medium, YE (0.01% *w*/*v*) and the same amount of PFASs mixture were added to 90 mL of water, while in another experimental set up, 90 mL of water were filter-sterilized (0.2 μm Filtropur; Sarstedt S.r.l., Trezzano sul Naviglio MI, Italy), being the filter used as inoculum.

All the enrichment cultures were incubated at 27 °C with shaking (150 rpm) under microaerophilic conditions for 3 weeks, being the cultures re-inoculated (1% *v*/*v*) at the end of every week in fresh media, enhancing the selective pressure derived by organofluorides on the biota prior the actual microbial isolation.

### 2.2. Microbial Isolation

After 3 weeks of enrichment cultures in the presence of the PFAS mixture, aliquots (100 μL) of the selected biota were serially diluted in sterile physiological solution (sodium chloride [NaCl] 0.9% *w*/*v*), and spread out onto 3 different solid media, where bacteria were recovered for 5 days at 27 °C under static mode, followed by their isolation. The media applied in this study were (i) plate count agar [PCA; composed (g L^−1^) of tryptone, 5; YE, 2.5; glucose, 1], (ii) Reasoner’s 2A agar [R2A; containing (g L^−1^) tryptone, 0.25; YE, 0.5; glucose, 0.5; peptone, 0.5; soluble starch, 0.5; casamino acids, 0.5; sodium pyruvate, 0.3; dipotassium phosphate (K_2_HPO_4_), 0.3; magnesium sulfate heptahydrate (MgSO_4_·7H_2_O), 0.024], and (iii) Waksman’s glucose agar [composed (g L^−1^) of peptone, 5; beef extract, 5; NaCl, 5; glucose, 10]. The pH values of the media were adjusted at ca. 7, being 15 g L^−1^ of Agar added when needed. PCA and R2A media were chosen due to their regular application in the enumeration and identification of microorganisms in potable water, wastewater, food, and dairy products in laboratory settings, while Waksman’s glucose was firstly described for screening actinomycete strains. All the reagents were purchased from Sigma-Aldrich^®^ (Merck Life Science S.r.l.).

### 2.3. Bacterial Strains Identification

The bacterial strains named as PS27 and PDMF10 strains, as well as all the obtained bacterial isolates, were identified within the microbial community harbored in the soil and groundwater samples contaminated by PFASs, respectively. The chromosomal DNA was extracted and purified through the chloroform-phenol method applied on biomasses grown for 24 h at 27 °C with shaking (150 rpm) in the liquid-rich medium Luria-Bertani [LB; composed (g L^−1^) of NaCl, 10; tryptone, 10; YE, 5]; when needed, LB was solidified by adding 15 g L^−1^ of Agar. The 16S rRNA gene product was obtain to identify at genus level the environmental isolates under investigation by using the universal primers F1 and R12 [24] under the following thermocycler conditions: (i) initial denaturation temperature of 95 °C for 5 min, (ii) run of 30 cycles with each cycle consisting of 30 s at 95 °C (denaturation), 30 s at 50 °C (annealing) and 1 min at 72 °C (extension), and (iii) final extension step at 72 °C for 10 min. The PCR product was cloned into the commercial plasmid pGEM^®^ T-Easy Vector (Promega Italia S.r.l., Milan, Italy), which was then sequenced (Eurofins Genomics S.r.l., Vimodrone MI, Italy). The sequence output was analyzed by using EZ Taxon-E [25] database. The 16S rRNA gene sequences of PS27 and PDMF10 strains were registered as accession MN861077 and MN861078 in the GenBank database, respectively.

### 2.4. Culture Conditions of PS27 and PFMF10 as Either Axenic Cultures (i.e., Growing or Resting Cells) or Mixed Culture

Before the inoculation of axenic cultures of PS27 and PDMF10 strains, these environmental isolates were pre-cultured for 24-h in sterile conical tubes (Sarstedt S.r.l., Trezzano sul Naviglio MI, Italy) containing 5 mL of LB medium at 27 °C with shaking (150 rpm). The day after, bacterial cells were inoculated (1% *v*/*v*) under metabolically controlled conditions [26] in 250 mL Erlenmeyer flasks containing 50 mL of DM medium amended with different initial concentrations organic solvents as only carbon and energy sources, which included: (i) octanol (2 mM), (ii) octane (61.5 mM), or (iii) a mixture [1:1 ratio (*v*/*v*)] of ethanol:octane (17.4 mM:6 mM), and in the presence/absence of PFHxS (50 μM). The bacterial growth was performed for either 5 or 15 (when PFHxS was added along with organic solvents) days. The actual biomass yield was expressed as the average of the logarithm of the Colony Forming Unit per milliliter of culture (Log_10_ CFU mL^−1^) for each biological trial (*n* = 3) with standard deviation.

As for resting cells, PS27 and PDM10 strains were cultivated in DM medium amended with either octane or ethanol:octane mixture up to the exponential growth phase (48-h), after which bacterial cells were collected through centrifugation (5000 *g* for 10 min) and washed twice with phosphate buffer saline solution [PBS; composed (g L^−1^) of NaCl, 8; KCl, 0.2; Na_2_HPO_4_, 1.44; KH_2_PO_4_, 0.24] at pH 7.4. The obtained resting cells were then re-suspended in 50 mL of fresh PBS amended with PFHxS (50 μM).

The mixed culture was generated by inoculating 250 μL of each pre-cultured strain (i.e., PS27 and PDMF10) to get a 1% *v*/*v* of biomass content in 50 mL of DM medium containing either octane or ethanol:octane mixture alongside PFHxS, with equal concentrations as in the case of single-species cultures. All the bacterial cultures were incubated at 27 °C with shaking (150 rpm).

Aliquots (500 μL) of the cell-free spent medium from of each culturing condition above-described, as well as those of the abiotic control cultures (consisting of: either DM medium or PBS [the latter in the case of resting cells experiments], organic solvents [only in the case of growing bacterial cells], PFHxS, and autoclaved [121 °C, 1 atmosphere of pressure, for 20 min] biomass), were sampled at the incubation start time (0) and after 5, 10, and 15 days of PFHxS exposure, in order to evaluate the biotic PFHxS removal efficiency as function of the different bacterial cell physiology tested. All the organic solvents and PFHxS were purchased from Sigma-Aldrich^®^ (Merck Life Science S.r.l.).

### 2.5. Liquid Chromatography Mass Spectrometry (LC-MS/MS)

The residual level of PFHxS (reported as μM concentration with standard deviation) per given microbial exposure time in all the bacterial cell-free spent medium and abiotic samples collected were measured via LC-MS/MS with the isotope dilution analysis technique. The liquid chromatograph was equipped with a Shimadzu Nexera X2 LC-30AD unit (Kyoto, Japan) coupled with SCIEX 6500 mass spectrometer (Framingham, MA, USA). The aqueous solution containing 6 mM ammonium formate (Sigma-Aldrich^®^, Merck Life Science S.r.l.) and methanol (Merck Serono S.p.A. Rome, Italy) as mobile phases were delivered at a flow rate of 300 μL min^−1^. Sample volume (100 µL) was injected onto a Supelco Ascentis Express RP-Amide 150 cm × 2.1 mm, 2.7 µm analytical column. Each sample was opportunely sonicated, diluted, and spiked with a mass-labeled internal standard before analysis. PFHxS was monitored for two transitions using scheduled MRM operating in negative ESI mode. A seven-point linear IS calibration curve was generated in the range 0.25–100 µM with calibration standards prepared in a 95:5 methanol/water solution. Native PFHxS standard and [1,2,3-^13^C_3_]PFHxS as Isotopic Labelled Internal Standard (ILIS) were purchased from Wellington Laboratories Inc. (Chemical Research 2000 S.r.l., Rome, Italy).

### 2.6. Transmission Electron Microscopy (TEM)

Bacterial cells grew up to the late exponential growth phase (72-h) under ethanol:octane (PS27) or octane (PDMF10) conditioning, and in the presence/absence of 50 μM PFHxS were collected, thin sectioned and imaged through Philips CM100 TEM operating at 80 kV, as described by Presentato and co-workers (2018) [27]. Briefly, the bacterial cells were fixed for 2-h at room temperature (RT) with a primary fixative solution composed of: glutaraldehyde (2.5% *v*/*v*), HEPES (50 mM) and magnesium chloride (MgCl_2_; 1 mM) at pH7, being successively rinsed 3 times with sodium cacodylate buffer (0.1 M) pH 7. After this step, the cells were further fixed with a secondary fixative solution consisting of osmium tetroxide (2% *v*/*v*) in sodium cacodylate buffer for 1-h at RT. The cells were then dehydrated by performing washes (3 times 15 min each) with increasing concentrations of water/ethanol solutions (30, 50, 70, 85, 95, and 100%), being finally washed (3 times 10 min each) with propylene oxide (>99.5%) prior cell infiltration over-night (16-h) at RT with a 1:1 mixture of propylene oxide:epoxy resin, which was prepared according to the manufacturer’s protocol. PS27 and PDMF10 cells were further infiltrated by epoxy resin (100%) for 4.5 h at RT, while resin polymerization occurred during incubations carried out at 45 and 60 °C for 12 and 24 h, respectively. The obtained resin blocks were ultrathin sectioned and mounted onto copper slot grids (CF300-CU, Electron Microscopy Sciences, Hatfield PA, USA). All the reagents to perform this procedure were purchased from Sigma-Aldrich^®^ (Merck Life Science S.r.l.).

## 3. Results and Discussion

Among the 79 bacterial isolates derived from the microbial community of environmental matrices contaminated with PFASs (Tables S1 and S2), the microbial strains PS27 (derived from soil) and PDFM10 (derived from groundwater) were chosen to establish if common mechanisms are exploited by microorganisms belonging to diverse environmental niches to cope with the presence of the fully fluorinated PFHxS in the cultivation medium. Particularly, the obtained 1515-bp (PS27) and 1412-bp (PDMF10) fragments of the 16S rRNA were sequenced and searched for nucleotide identity using EZ-Taxon database. This analysis revealed that PS27 shares 99% identity with *Pseudomonas humi* (LC145037) and *delhiensis* (jgi.1118306) species, while PDMF10 displayed 98% of identity with *P. alcaligenes* (BATI01000076); hence, these microorganisms were tentatively identified as belonging to the *Pseudomonas* genus.

**Figure 1:**
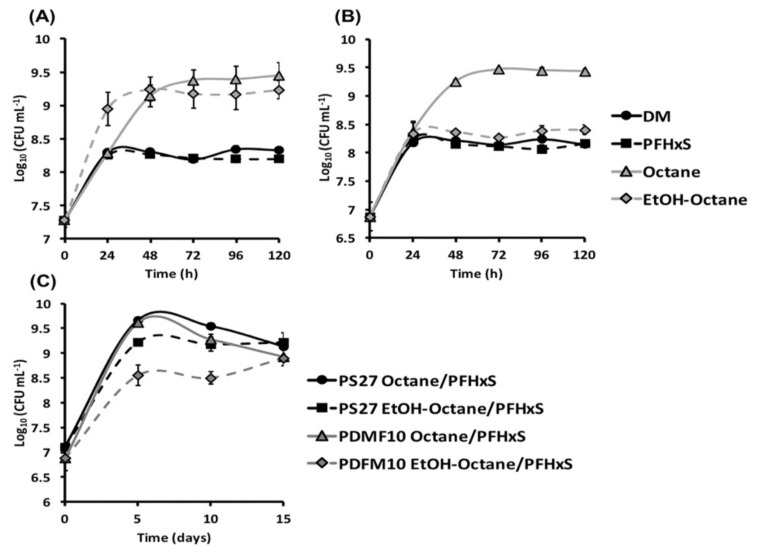
Growth profiles of PS27 (**A**) and PDMF10 (**B**) environmental isolates cultured in DM only or supplied with PFHxS, octane, or the mixture ethanol-octane as the only source of carbon and energy. In (**C**) is shown the trend of bacterial growth while the cells are incubated in DM amended with organic solvents alongside PFHxS.

Despite the increasing research interest and number of studies devoted to the detection of PFASs into the environment, there are still gaps to be filled regarding their origin, attenuation or, in the best-case scenario, actual removal. In this respect, Ellis and co-workers (2001) brought to light how the thermolysis of fluoropolymers can lead to the appearance of persistent organofluorides [28], while other investigations revealed that perfluorocarboxylic acids (PFCAs) can derive from the biotic transformation of fluorotelomer alcohols (FTOHs) [29]. Indeed, the adapted halogen-alkane and/or alcohol degrading cultures, mixed microbial cultures, activated sludge, and soils [29,30,31,32] were proficient in producing PFOA from FTOHs and, depending on the degree of defluorination, shorter-chain perfluoroalkyl acids, such as perfluorohexanecarboxylic acid (PFHA). Another important consideration to be made is that PFASs and related compounds do not serve as a source of carbon and energy to sustain bacterial growth. PS27 and PDMF10 do not represent an exception, since their growth profiles, when PFHxS was supplied to DM, were not any different from those obtained when bacterial cells were cultivated without PFHxS addition (Figure 1A,B).

**Figure 2:**
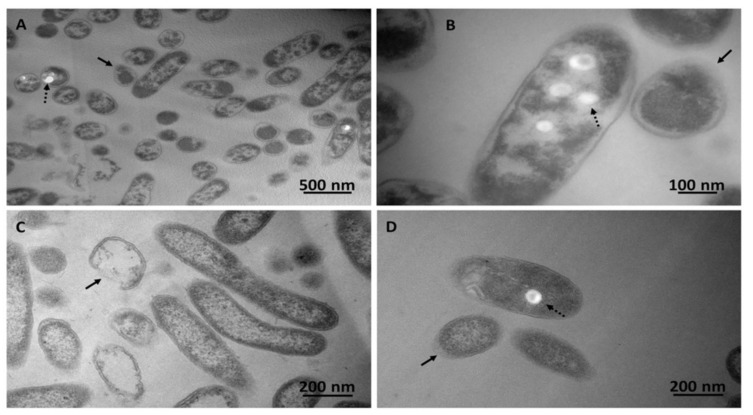
Transmission electron micrographs of PS27 (**A**,**B**) or PDMF10 (**C**,**D**) grown for 72-h in the presence of the mixture ethanol-octane or octane, respectively. Black arrows and dashed ones indicate cell structural features and electron transparent inclusion bodies, respectively.

Thus, to achieve a biotic transformation of these pollutants, an alternative carbon substrate is needed [29,33,34,35]. This aspect would likely lead microorganisms to elicit cometabolic reactions, where the primary substrate supporting bacterial growth will determine a specific biochemical asset, which can result in the biotic transformation of compounds (e.g., PFASs) that may or may not be a structural analog of the growth substrate. *Pseudomonas* strains are known for their alkanotrophic features, holding the ability to utilize a vast array of *n*-alkanes (from C_2_ to C_30_) for energetic purposes [36,37,38,39]. Indeed, the occurrence of enzymes like monooxygenases with a broad specificity of substrate binding [40] enables *Pseudomonas* spp. to transform complex pollutants as chlorinated alkanes and aromatics via cometabolic reactions [41,42,43]. However, the mere presence of toxic organic solvents can hamper the pollutant removal efficacy by bacteria, as these hydrophobic chemicals target mainly the cytoplasmic membrane, causing an increase of its fluidity, loss of its functionality, and ultimately cell death [44,45]. PS27 and PDMF10 cell growth under octanol, octane, or ethanol-octane pressure was not impaired by the most common fluidizing effect exerted by hydrophobic compounds upon biological membranes, as any lag phase was not observed over a period of 5 days; nevertheless, PDMF10 resulted less prone, yet efficient, to thrive in the presence of the ethanol-octane mixture as compared to PS27 (Figure 1A,B, Figure S1). Thus, these *Pseudomonas* spp. may counteract the disruptive effect of hydrophobic compounds by triggering short-term responses (i.e., *cis*-*trans* fatty acid isomerization) to readjust the lipid type content in their membranes, as described in the case of *P. putida* S12 exposed to either 1-octanol or 3-nitrotoluene [46,47]. However, TEM imaging revealed that both isolates presented, at the morphological level, the presence of the organic solvents (Figure 2).

For example, bacterial cells grew under ethanol-octane (PS27; Figure 2A,B) or octane (PDMF10; Figure 2C,D) stress featured round-shaped morphologies, other than the usual rod-shaped one. These changes may be caused by the high solvent concentration that represented a stress factor for *Pseudomonas* cells, as also suggested by a less electron-dense peripheral layer (indicated by black arrows), which might be caused by a loss of membrane integrity. These observations are in line with other studies where the membrane fluidity and permeability of *Pseudomonas* cells were compromised by excesses of ethanol [48]. Reasonably, the accumulation of lipophilic compounds between the acyl chains of the phospholipid bilayers led to the alteration of the membrane properties through a process known as narcosis [27,49], which can eventually determine swelling of the phospholipid bilayer, causing the transition towards round-shape morphology [50]. Further, both strains were capable of synthesizing electron transparent intracellular accumulations (Figure 2A–C indicated by black dashed arrows) likely containing hydrophobic storage compounds (e.g., polyhydroxyalkanoates), which were elsewhere reported to act as carbon and energy-reserve material by most microorganisms, since these storage compounds are produced by bacteria when they experience unbalanced nutritional conditions and/or environmental stresses [51,52]. The nature of these specialized lipids was not investigated any further, although the added value of these compounds is undisputed since they are the building block polymers for synthesizing *ecofriendly* and biodegradable plastics [53].

Overall, the presence of PFHxS alongside organic solvents did not negatively affect the growth of both isolates within 15 days of bacterial incubation (Figure 1C). Given that PFHxS can be considered structurally analog to the organic solvents utilized in this study to sustain bacterial growth, it can be expected that these isolates can use their enzymatic asset devoted to solvent oxidation also to transform PFHxS. Past studies have shown how microorganisms can defluorinate and mineralize the carbon bonds in PFASs, as well as how this biotic process was improved by replenishing the mixed culture with ethanol, however leading to the formation of shorter fluorinated carbon metabolites [30,31]. Moreover, *Pseudomonas* sp. D2, exploiting glucose and/or acetate as the only source of carbon and energy, partially degraded PFOS, highlighting that even non-related carbon substrates can elicit cometabolic reactions [33]. Other investigations unveiled that PFOA and PFOS can be decomposed by pure bacterial cultures of *P. parafulva*, *P. aeruginosa*, or *P. plecoglossicida*, being the latter capable of utilizing PFOS as carbon source biotransforming it in perfluoroheptanoic acid with the release of fluorine ions [54,55,56]. Further, Huang and Jaffé (2019) reported on the ability of *Acidimicrobium* sp. A6 strain to perform bioreductive defluorination of both PFOA and PFOS, using either ammonium or hydrogen as an electron donor [57]. From these examples, it is evident how the interaction between poly-fluorinated alkyl substances and microorganisms takes place, however, leading to the generation of short-chain fully fluorinated compounds for which any biotic reaction aimed to their breakdown is not described.

**Figure 3:**
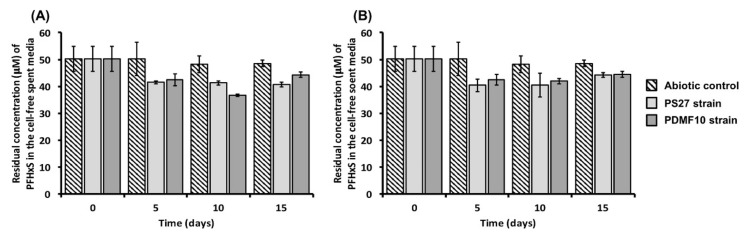
Removal efficacy of PFHxS performed by PS27 and PDMF10 growing cells under either octane (**A**) or ethanol-octane (**B**) conditioning.

This aspect is due to the strength and polarity of C-F bond, the perfluoroalkyl rigidity, and the absence of reactive groups in PFASs [3,58]; hence, once released into the environment, these chemicals will persist and endure. In the present study, PS27 strain was not able to transform PFHxS during 10 days of incubation in the presence of octanol as a growth substrate (Figure S2), similarly to *Pseudomonas* sp. OCY4, which, yet capable of cometabolic transform 8:2 FTOH under octanol conditioning, determined the formation of short-chain fluorinated acids (i.e., PFHA) that were not further degraded [34]. This “side effect” was also highlighted in *P. butanovorans* and *P. oleovorans* cultures, where alkanes and/or alcohols were added to the growth medium to achieve the cometabolic transformation of FTOHs, being PFOA and PFHxA identified as both major and stable metabolic products derived from 6:2 FTOH or 8:2 FTOH [35], respectively. Here, a change of the carrier solvent (i.e., octane and ethanol-octane mixture) determined a certain degree of PFHxS bioaccumulation (Figure 3), which may be a collateral effect exerted by the greater hydrophobicity of octane as compared to the corresponding alcohol (i.e., octanol). Indeed, bacterial cells can produce biosurfactants as emulsifying agents to enhance the water solubility of the highly hydrophobic substrate of growth (e.g., *n*-alkane), which is scarcely bioavailable in the broth culture, and, therefore, its uptake within the cells [59]. This bacterial physiological adaptation to the organic solvents could reasonably explain the biotic accumulation of PFHxS, as under octane conditioning PS27 could remove 16 ± 2% of PFHxS after 5 days of incubation, being no further accumulation detected within 15 days of monitoring. Notably, PDMF10 showed the highest extent of PFHxS accumulation (24 ± 1%) after 10 days (Figure 3A). On the other hand, 5 days of incubation of both strains in the presence of the mixture ethanol-octane were needed to reach the maximum yield of PFHxS accumulation, being 24 ± 5% (PS27) and 15 ± 1% (PDMF10) (Figure 3B).

**Figure 4:**
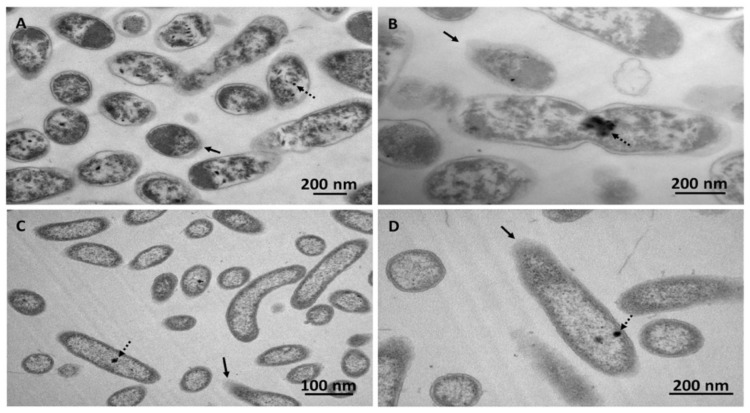
Transmission electron micrographs of PS27 (**A**,**B**) or PDMF10 (**C**,**D**) grown for 72 h in the co-presence of PFHxS and the mixture ethanol-octane or octane, respectively. Black arrows and dashed ones indicate cell structural features and electron-dense accumulation, respectively.

**Figure 5:**
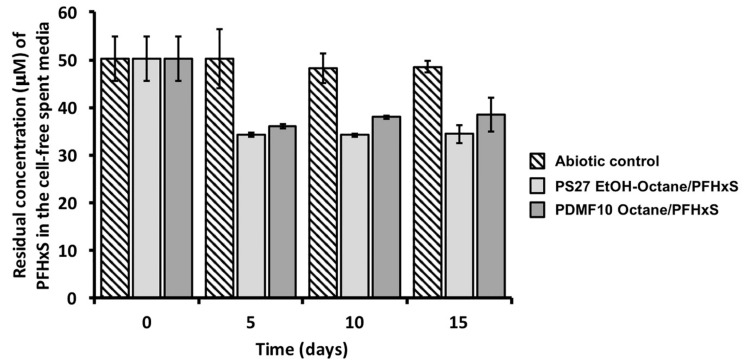
Removal efficacy of PFHxS performed by PS27 and PDMF10 resting cells under ethanol-octane and octane conditioning, respectively.

The biotic accumulation of PFHxS was further supported by the appearance of small sphere-shaped electron-dense focuses (Figure 4 indicated by black dashed arrows) within PS27 and PDMF10 cells grown under ethanol-octane and octane pressure, respectively. Moreover, bacterial cells grown in the presence of PFHxS shared similar morphological features (i.e., round-shape and damaged membrane) with PFHxS-free biomasses (Figure 2). The only difference relied on the absence of electron transparent inclusion bodies, most likely due to their mobilization because of the further stress exerted by PFHxS along with organic solvents (Figure 4). Regardless the amount of PFHxS accumulated by these strains, a common feature was the release of PFHxS during 15 days of incubation; indeed, the amount of PFHxS removed dropped down to ca. 10% (Figure 3) due to cellular turn over. Since PS27 and PDMF10 showed the best PFHxS accumulation performances under ethanol-octane or octane growth conditions respectively, the corresponding metabolically active, yet not growing, resting cells were analyzed to unveil their accumulative potential as function of a different physiological state. As a result, 32 ± 1% and 28 ± 1% of PFHxS were removed by ethanol-octane- (PS27) or octane-adapted (PDMF10) resting cells within 5 days of bacterial exposure (Figure 5), likely due to a higher biomass availability per given time able to cope with PFHxS as compared to growing cells. Nevertheless, a partial release of PFHxS accumulated was observed at the latest stage (15 days) of resting cell exposure, being 29 ± 4% and 21 ± 7% the actual amount of PFHxS removed by PS27 and PDMF10, respectively (Figure 5).

**Figure 6:**
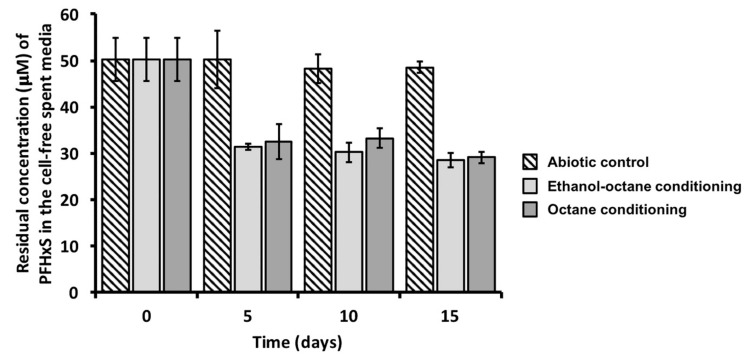
Removal efficacy of PFHxS performed by the mixed microbial culture under either ethanol-octane or octane conditioning.

Finally, an even enhanced PFHxS accumulative power (40 ± 3%) was detected when the two bacterial isolates were tested as mixed culture (Figure 6), highlighting a synergistic effect of PS27 and PDMF10 in removing the short-chain fluorinated acid, with no evidence of its release during the timeframe tested.

## 4. Conclusions

The present study provides novel information on the interaction between bacteria and the emergent class of hazardous perfluorinated chemicals, focusing the attention on short-chain PFAS (i.e., PFHxS). The latter often represents the end biotransformation product of longer PFASs, which are hard to degrade through biotic and/or physical-chemical approaches. Thus, naturally occurring microorganisms isolated from the indigenous microbiota inhabiting environmental matrices (i.e., soil and groundwater) contaminated with PFASs were tested for their ability to handle the recalcitrant PFHxS. Upon an opportune selective pressure, PS27 and PDMF10 environmental isolates could accumulate PFHxS within the cells, being proficient in counteracting the stress derived from this compound under alkanotrophic conditions. The accumulative behavior of these strains improved as function of the bacterial physiology investigated; metabolically active, yet not growing, resting cells were more prone to remove PFHxS than growing ones, although the mixed microbial culture revealed the best accumulative performance within a relative short period (5 days) of incubation. Considering (i) the alarming increasing level of short-chain PFASs into the environment, (ii) their enhanced recalcitrance to degradation, (iii) the absence of biological catalysts capable of completely decomposing these short-chain fluorochemicals, and (iv) the inefficiency of physical-chemical strategies (e.g., injectable particulate carbon or granular activated carbon) for their removal [60], these results are of some importance, in light of the possibility to merge existing technologies with the unveiled accumulative potential of the bacterial isolates here investigated.

## Supplementary Materials

The following are available online at https://www.mdpi.com/2076-2607/8/1/92/s1, Table S1: Bacterial strains isolated from the microbial community inhabiting the soil contaminated by PFASs, Table S2: Bacterial strains isolated from the microbial community inhabiting the groundwater contaminated by PFASs, Figure S1: Growth profile of PS27 strain in the presence of either 1-octanol or in the co-presence of the organic solvent alongside PFHxS, Figure S2: Removal efficacy of PFHxS performed by PS27 growing cells under 1-octanol conditioning.
