# Supplementary material for: On the Ability of Perfluorohexane Sulfonate (PFHxS) Bioaccumulation by Two Pseudomonas sp. Strains Isolated from PFAS-Contaminated Environmental Matrices
Source: Microorganisms. 2020 Jan 9;8(1):92. doi: 10.3390/microorganisms8010092 (PMC7022908; doi:10.3390/microorganisms8010092)
Supplement: Supplementary file 1 [file microorganisms-08-00092-s001.pdf]

Supplementary information

On the ability of perfluorohexane sulfonate (PFHxS) bioaccumulation by two *Pseudomonas* sp. strains isolated from PFAS-contaminated environmental matrices

Alessandro Presentato [1], Silvia Lampis [2,*], Andrea Vantini [3], Flavio Manea [3], Francesca Daprà [3], Stefano Zuccoli [2], Giovanni Vallini [2]

[1] Department of Biological, Chemical and Pharmaceutical Sciences and Technologies (STEBICEF) – University of Palermo, Italy; alessandro.presentato@unipa.it
[2] Department of Biotechnology – University of Verona, Italy; silvia.lampis@univr.it; stefano.zuccoli@studenti.univr.it; giovanni.vallini@univr.it
[3] Regional Agency for Environmental Prevention and Protection of Veneto (ARPAV) – Regional Laboratories, Verona, Italy; andrea.vantini@arpa.veneto.it; flavio.manea@arpa.veneto.it; francesca.dapra@arpa.veneto.it

* Correspondence: silvia.lampis@univr.it

Table S1: bacterial strains isolated from the microbial community inhabiting the soil contaminated by PFASs.

Bacterial isolates	Identity (%)	Type reference strains	Accession number	Taxonomy
RS11	99	*Pseuodomonas delhiensis*	JGI.1118306	Gammaproteobacteria
RS12	99	*Pseudomonas veronii*	JYLL01000074	Gammaproteobacteria
RS15	99	*Enterobacter ludwigii*	JTLO01000001	Gammaproteobacteria
RS16	99	*Pseudomonas veronii*	JYLL01000074	Gammaproteobacteria
RS17	98	*Pseuodomonas delhiensis*	JGI.1118306	Gammaproteobacteria
RS18	99	*Bordetella petrii*	AM902716	Betaproteobacteria
RS21	99	*Pseuodomonas extremaustralis*	AHIP01000073	Gammaproteobacteria
RS23	99	*Pseuodomonas aeruginosa*	BAMA01000316	Gammaproteobacteria
RS25	99	*Pseudomonas veronii*	JYLL01000074	Gammaproteobacteria
RS26	99	*Starkeya novella*	CP002026	Alphaproteobacteria
RS31	98	*Citrobacter farmeri*	AF025371	Gammaproteobacteria
RS33	99	*Achromobacter dolens*	HF586509	Betaproteobacteria
RS34	99	*Citrobacter farmeri*	AF025371	Gammaproteobacteria
RS36	99	*Bordetella petrii*	AM902716	Betaproteobacteria
RS37	99	*Achromobacter insolitus*	CP019325	Betaproteobacteria
PS11	98	*Pseudomonas nitrireducens*	HM246143	Gammaproteobacteria
PS12	99	*Pseudomonas simiae*	AJ936933	Gammaproteobacteria
PS13	99	*Enterobacter ludwigii*	JTLO01000001	Gammaproteobacteria
PS16	99	*Starkeya novella*	CP002026	Alphaproteobacteria
PS17	99	*Starkeya novella*	CP002026	Alphaproteobacteria
PS18	100	*Pseuodomonas delhiensis*	JGI.1118306	Gammaproteobacteria
PS21	99	*Pseudomonas veronii*	JYLL01000074	Gammaproteobacteria
PS23	99	*Citrobacter farmeri*	AF025371	Gammaproteobacteria
PS25	98	*Citrobacter farmeri*	AF025371	Gammaproteobacteria
PS27	99	*Pseudomonas humi; Pseuodomonas delhiensis*	LC145037 JGI.1118306	Gammaproteobacteria
PS28	99	*Starkeya novella*	CP002026	Alphaproteobacteria
PS29	98	*Starkeya novella*	CP002026	Alphaproteobacteria
PS33	99	*Achromobacter insolitus*	CP019325	Betaproteobacteria
PS34	99	*Pseuodomonas delhiensis*	JGI.1118306	Gammaproteobacteria
PS35	99	*Pseudomonas veronii*	JYLL01000074	Gammaproteobacteria
PS38	99	*Starkeya novella*	CP002026	Alphaproteobacteria
PS39	99	*Starkeya novella*	CP002026	Alphaproteobacteria
WS11	99	*Serratia proteamaculans*	AJ233434	Gammaproteobacteria
WS12	99	*Pseudomonas veronii*	JYLL01000074	Gammaproteobacteria
WS15	99	*Pseudomonas simiae*	AJ936933	Gammaproteobacteria
WS16	99	*Enterobacter ludwigii*	JTLO01000001	Gammaproteobacteria
WS22	99	*Enterobacter ludwigii*	JTLO01000001	Gammaproteobacteria
WS25	94	*Rhodanobacter rhizosphaerae*	FJ772032	Gammaproteobacteria
WS31	99	*Serratia liquefaciens*	CP006252	Gammaproteobacteria
WS32	99	*Enterobacter ludwigii*	JTLO01000001	Gammaproteobacteria

Table S2: bacterial strains isolated from the microbial community inhabiting the groundwater contaminated by PFASs.

Bacterial isolates	Identity (%)	Type reference strains	Accession number	Taxonomy
RDM2	99	*Comamonas thiooxydans*	BBVD01000034;	Betaproteobacteria
RDM11	99	*Variovorax bronicumulans*	AB300597	Betaproteobacteria
RDM14	99	*Acidovorax soli*	JGI.1085893	Betaproteobacteria
RDMF2	99	*Aeromonas taiwansis*	CDDD01000060	Gammaproteobacteria
RDMF5	93	*Pseudomonas marginalis*	AJ308309	Gammaproteobacteria
RDMF10	97	*Pseudomonas granadiensis*	LT629778	Gammaproteobacteria
RDMF14	98	*Albidiferax ferrireducens*	CP000267	Betaproteobacteria
RDMF15	99	*Pseudomonas gessardii*	MNPU01000117	Gammaproteobacteria
RDMYE2	99	*Aeromonas caviae*	CDBK01000019	Gammaproteobacteria
RDMYE6	99	*Aeromonas taiwansis*	CDDD01000060	Gammaproteobacteria
RDMYE7	97	*Pseudomonas baetica*	FM201274	Gammaproteobacteria
RDMYE8	92	*Aeromonas caviae*	CDBK01000019	Gammaproteobacteria
PDM1	99	*Pseudomonas veronii*	JYLL01000074	Gammaproteobacteria
PDM2	98	*Acinetobacter haemolyticus*	APQQ01000002	Gammaproteobacteria
PDM10	99	*Variovorax boronicumulans*	AB300597	Betaproteobacteria
PDM11	93	*Flavobacterium tilapiae*	HQ111525	Flavobacteria
PDMF1	99	*Pseudomonas nitrireducens*	HM246143	Gammaproteobacteria
PDMF2	99	*Citrobacter amalonaticus*	FR870441	Gammaproteobacteria
PDMF3	99	*Pseudomonas donghuensis*	AJJP01000212	Gammaproteobacteria
PDMF7	99	*Pseudomonas marginalis*	AJ308309	Gammaproteobacteria
PDMF9	99	*Flavobacterium resistens*	EF575563	Flavobacteria
PDMF10	98	*Pseudomonas alcaligenes*	BATI01000076	Gammaproteobacteria
PDMF11	99	*Pseudoxanthomonas japonensis*	AB008507	Gammaproteobacteria
PDMYE1	99	*Pseudomonas mosselii*	AF072688	Gammaproteobacteria
PDMYE5	99	*Pseudomonas alkylphenolica*	CP009048	Gammaproteobacteria
PDMYE8	99	*Aeromonas media*	CDBZ01000012	Gammaproteobacteria
WDM8	99	*Pseudomonas veronii*	JYLL01000074	Gammaproteobacteria
WDMF2	99	*Delftia acidovorans*	JOUB01000005	Betaproteobacteria
WDMF6	99	*Pseudomonas saponiphila*	FNTJ01000001	Gammaproteobacteria
WDMF7	99	*Pseudomonas protegens*	CP003190	Gammaproteobacteria
WDMF8	99	*Pseudomonas protegens*	CP003190	Gammaproteobacteria
WDMF10	99	*Rhizobium oryziradicis*	KX129901	Alphaproteobacteria
WDMF11	99	*Rhodanobacter thiooxydans*	AJXW01000099	Gammaproteobacteria
WDMF12	98	*Shinella kummerowiae*	EF070131	Alphaproteobacteria
WDMYE1	98	*Bacillus proteolyticus*	MACH01000033	Bacilli
WDMYE3	99	*Delftia lacustris*	JGI.1102360	Betaproteobacteria
WDMYE4	98	*Acinetobacter haemolyticus*	APQQ01000002	Gammaproteobacteria
WDMYE7	98	*Aeromonas salmonicida*	LSGW01000109	Gammaproteobacteria
WDMYE8	94	*Bacillus gaemokensis*	LTAQ01000012	Bacilli

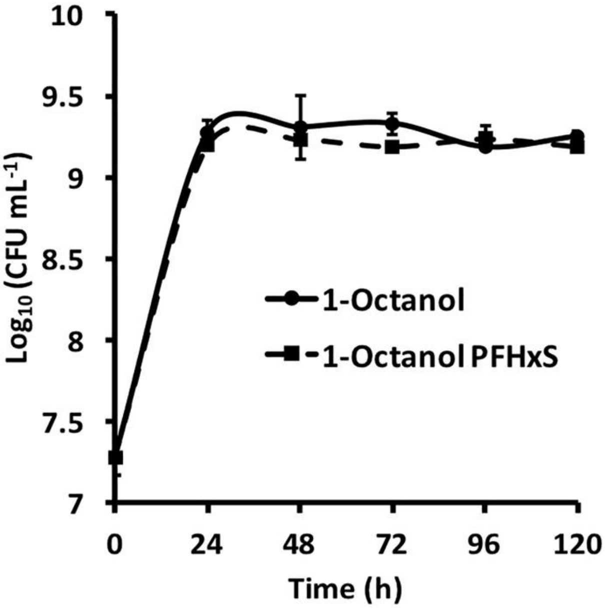

Figure S1: Growth profile of PS27 strain in the presence of either 1-octanol or in the co-presence of the organic solvent alongside PFHxS.

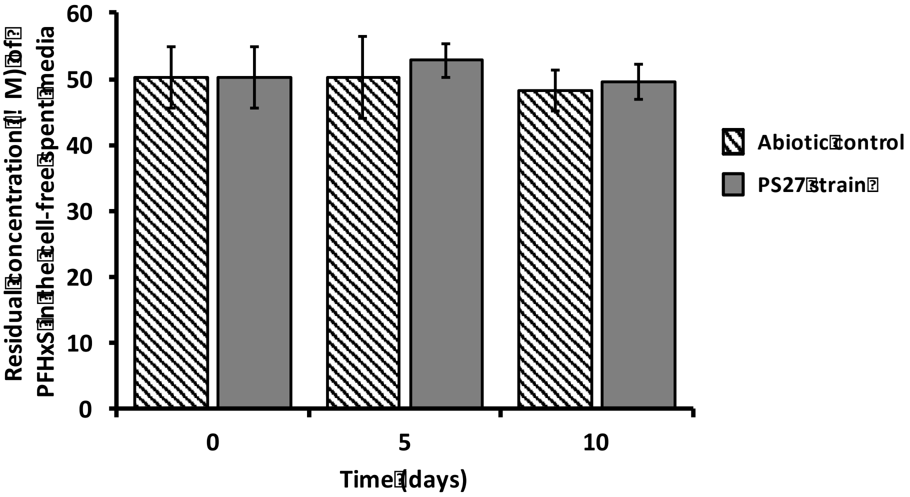

Figure S2: Removal efficacy of PFHxS performed by PS27 growing cells under 1-octanol conditioning.
